# Optimizing Promoters and Subcellular Localization for Constitutive Transgene Expression in *Marchantia polymorpha*

**DOI:** 10.1093/pcp/pcae063

**Published:** 2024-06-01

**Authors:** Sze Wai Tse, Davide Annese, Facundo Romani, Fernando Guzman-Chavez, Ignacy Bonter, Edith Forestier, Eftychios Frangedakis, Jim Haseloff

**Affiliations:** Department of Plant Sciences, University of Cambridge, Cambridge CB2 3EA, UK; Department of Plant Sciences, University of Cambridge, Cambridge CB2 3EA, UK; Department of Plant Sciences, University of Cambridge, Cambridge CB2 3EA, UK; Department of Plant Sciences, University of Cambridge, Cambridge CB2 3EA, UK; CONAHCyT, Instituto de Investigaciones Biomédicas, Universidad Nacional Autónoma de México (UNAM), CDMX 04510, México; Department of Plant Sciences, University of Cambridge, Cambridge CB2 3EA, UK; Department of Plant Sciences, University of Cambridge, Cambridge CB2 3EA, UK; Department of Plant Sciences, University of Cambridge, Cambridge CB2 3EA, UK; Department of Plant Sciences, University of Cambridge, Cambridge CB2 3EA, UK

**Keywords:** Betalain, *Marchantia polymorpha*, Metabolic engineering, Promoter, Recombinant protein, Synthetic biology

## Abstract

*Marchantia polymorpha* has become an important model system for comparative studies and synthetic biology. The systematic characterization of genetic elements would make heterologous gene expression more predictable in this test bed for gene circuit assembly and bioproduction. Yet, the toolbox of genetic parts for *Marchantia* includes only a few constitutive promoters that need benchmarking to assess their utility. We compared the expression patterns of previously characterized and new constitutive promoters. We found that driving expression with the double enhancer version of the cauliflower mosaic virus 35S promoter (_pro_35S × 2) provided the highest yield of proteins, although it also inhibits the growth of transformants. In contrast, promoters derived from the Marchantia genes for ETHYLENE RESPONSE FACTOR 1 and the CLASS II HOMEODOMAIN-LEUCINE ZIPPER protein drove expression to higher levels across all tissues without a growth penalty and can provide intermediate levels of gene expression. In addition, we showed that the cytosol is the best subcellular compartment to target heterologous proteins for higher levels of expression without a significant growth burden. To demonstrate the potential of these promoters in Marchantia, we expressed *RUBY*, a polycistronic betalain synthesis cassette linked by P2A sequences, to demonstrate coordinated expression of metabolic enzymes. A heat-shock-inducible promoter was used to further mitigate growth burdens associated with high amounts of betalain accumulation. We have expanded the existing tool kit for gene expression in Marchantia and provided new resources for the Marchantia research community.

## Introduction

Promoters play a key role in controlling the levels and cell specificity of gene expression. In plants, native, heterologous and synthetic promoters (Villao-Uzho et al. 2023), have been characterized for their ability to drive higher levels of expression, primarily in transient expression systems such as *Nicotiana benthamiana* (Engler et al. 2014, Brückner et al. 2015, Cai et al. 2020, Tian et al. 2022). However, studies rarely describe native promoters for stable transformation, and the majority focus on the model species *Arabidopsis thaliana* (Holtorf et al. 1995, Han et al. 2015, Jiang et al. 2018, Yang and Nemhauser 2023). As expression levels associated with promoters are not easily transferable between plant species, the lack of promoter characterization in non-model systems has become a limitation for the design and assembly of genetic circuits.

*Marchantia polymorpha*, an emerging land plant model (Bowman et al. 2022), is ideally suited to address this shortcoming, thanks to its short cycle of growth and reproduction, remarkable regenerative capacity and the extensive set of available experimental tools (Ishizaki et al. 2015, Sauret-Güeto et al. 2020). Marchantia also produces clonal offspring called gemmae, making it suitable for large-scale comparative studies due to the ease of imaging at a cellular level and rapid growth. Additionally, nuclear transformation can be performed on a large scale, enabling the generation of hundreds of lines within 3–4 weeks, in contrast to systems like *A. thaliana* or *N. benthamiana*, which may take months (Ishizaki et al. 2008, 2015, Sauret-Güeto et al. 2020, Romani et al. 2024). The Marchantia system has potential as a platform for facile testing of promoters for genetic and biotechnological applications.

A number of studies have explored Marchantia as a platform for the production of recombinant proteins (Frangedakis et al. 2021) and metabolites (Takemura et al. 2013), but comparative studies of constitutive promoters in Marchantia are scarce (Althoff et al. 2014). Such work is necessary because some heterologous promoters in Marchantia do not perform as expected. For instance, the widely used cauliflower mosaic virus 35S promoter (_pro_35S) showed a non-homogenous expression pattern in Marchantia (Amack and Antunes 2020), as previously seen in other bryophyte species such as *Physcomitrium patens* (Finka et al. 2008, Saidi et al. 2009), while the *Agrobacterium tumefaciens Nopaline Synthase* promoter (_pro_*NOS*) is poorly functional in Marchantia (Ishizaki et al. 2015). In addition, Marchantia promoters do not necessarily perform well in transformed *N. benthamiana* leaves and BY2 suspension cell cultures (Tian et al. 2022), confirming that promoter activity cannot be reliably extrapolated between divergent plants such as angiosperms and liverworts.

Four promoters with broad patterns of expression are commonly used in Marchantia. The native *ELONGATION FACTOR 1*α promoter (_pro_Mp*EF1*α) (Althoff et al. 2014), the single and double enhancer versions of the cauliflower mosaic virus 35S promoter [_pro_*35S* (Althoff et al. 2014) and _pro_*35S × 2* (Ishizaki et al. 2015)] and the Marchantia UBIQUITIN-CONJUGATING ENZYME E2 promoter (_pro_Mp*UBE2*) (Sauret-Güeto et al. 2020). It was shown before that _pro_Mp*EF1α* can drive the expression of high levels of recombinant protein but its expression pattern is stronger near the apical notch, while the single and double enhancer versions of the cauliflower mosaic virus 35S promoter show comparatively weaker expression near the apical notch (Althoff et al. 2014, Ishizaki et al. 2015). As an alternative, _pro_Mp*UBE2* showed a more even expression pattern across the gemma (Sauret-Güeto et al. 2020). A set of five promoters was released as part of the OpenPlant tool kit (Sauret-Güeto et al. 2020) as synthetic parts compatible with modular cloning (Patron et al. 2015) but systematic comparison is lacking. Recently, a large set of promoters derived from transcription factors in Marchantia has been published (Romani et al. 2024), providing a new pool of candidates for selection of promoters that drive strong and ubiquitous expression.

Besides promoter selection, maximizing expression can be achieved by targeting the protein to specific compartments such as the apoplast, chloroplast and endoplasmic reticulum (ER) (Feng et al. 2022, Liu and Timko 2022), but this strategy remains largely unexplored in Marchantia. During transient expression in *N. benthamiana*, targeting to the apoplast was reported to produce human growth hormone at levels up to 10% of total soluble protein (Gils et al. 2005), and anti-hen egg white lysozyme nanobody up to 30% of the total leaf protein (Teh and Kavanagh 2010). Furthermore, post-translational targeting to the chloroplast yielded 0.1–11% of recombinant proteins in various systems (Muthamilselvan et al. 2019). As for targeting to the ER, the formation of protein bodies induced by high levels of accumulation of recombinant proteins can further boost the yield up to 40–50% of total soluble content (Bankar et al. 2009, Saberianfar et al. 2015). Transgene expression from the Marchantia chloroplast genome produced up to 400–500 µg/g (15% of total soluble protein) FW of mTurquoise2 fluorescent protein (Frangedakis et al. 2021), but yields of protein expressed from the nuclear genome, which allows for diverse subcellular targeting, remain poorly characterized.

We characterized the relative strengths of a collection of nine putative constitutive promoters, four from the OpenPlant tool kit and five native promoters derived from the transcription factors. We compared levels of accumulation for proteins with different subcellular targeting, the first such data for Marchantia. Furthermore, we also tested the expression of the betalain biosynthesis cassette RUBY (He et al. 2020) and quantified the yield of betanin. This provided quantitative characterization of promoters that will be useful for gene circuit assembly, and a demonstration of the Marchantia system as a test bed for metabolic engineering and yield benchmarks.

## Results

### Expression patterns of constitutive promoters

**Fig. 1:**
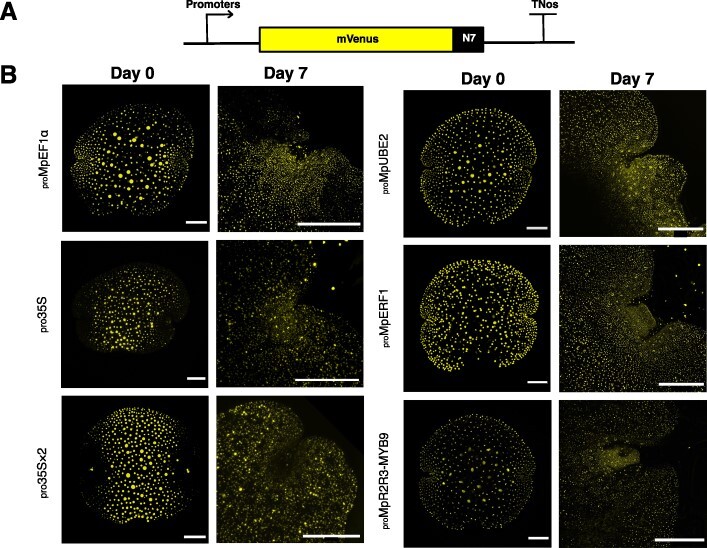
Expression pattern of mVenus-N7 reporter driven by different promoters in day 0 *Marchantia* gemmae and day 7 plants from different promoter:mVenus-N7 lines. (A) Schematic representation of the promoter:mVenus-N7 constructs. (B) Confocal microscopy Images, mVenus-N7 signal shown light coloured. Scale bar for day 0 plants = 100 µm. Scale bar for day 7 plants = 400 µm. Gene IDs: Mp*ERF1* (Mp1g20040), *R2R3-MYB TRANSCRIPTION FACTOR 9* (*R2R3-MYB9*, Mp5g14610), *ELONGATION FACTOR 1α* (Mp*EF1α*, Mp3g23400) and Mp*UBE*2 (Mp5g21800).

We aimed to develop novel constitutive promoter elements for Marchantia, which would allow high levels of transgene expression as well as exhibit homogenous expression throughout the thallus. We examined a collection of ∼400 putative transcription factor promoter regions fused to the mVenus-N7 reporter (**Fig. 1A**) (Romani et al. 2024). We screened for promoters that exhibited bright mVenus fluorescence across all tissue types in day 0 gemmae and day 7 plants. This allowed us to pick five candidates: Marchantia *ETHYLENE RESPONSE FACTOR 1* (_pro_Mp*ERF1*), _pro_Mp*ERF4*, _pro_Mp*C2HDZ*, _pro_Mp*1R-MYB7* and _pro_Mp*R2R3-MYB9*.

We first compared the expression patterns of the five candidates with _pro_*35S* and _pro_*35S × 2*, _pro_Mp*Ef1*α and _pro_Mp*UBE2* reporter fusions in day 0 gemmae and day 7 plants. These candidates were selected as they all appeared to drive expression of mVenus-N7 across all tissue types and strongly during a preliminary screening of day 0 gemmae. Consistent with previously reported data (Althoff et al. 2014), for all time points, _pro_*35S* and _pro_*35S × 2* plants showed low expression in the cells close to the apical notch (**Fig. 1B**), in contrast to _pro_Mp*Ef1*α, where the strongest expression was observed in cells close to the apical notch. Expression of the _pro_Mp*UBE2*-driven reporter was ubiquitous across all tissue types from gemmae at day 0 to day 7 plants, as it was for _pro_Mp*ERF1* and _pro_Mp*R2R3-MYB9* (**Fig. 1B**). The expression patterns of _pro_Mp*1R-MYB7*, _pro_Mp*C2HDZ* and _pro_Mp*ERF4* reporters were also ubiquitous across all tissue types (Supplementary Fig. S1).

### Benchmarking promoters for heterologous expression

**Fig. 2:**
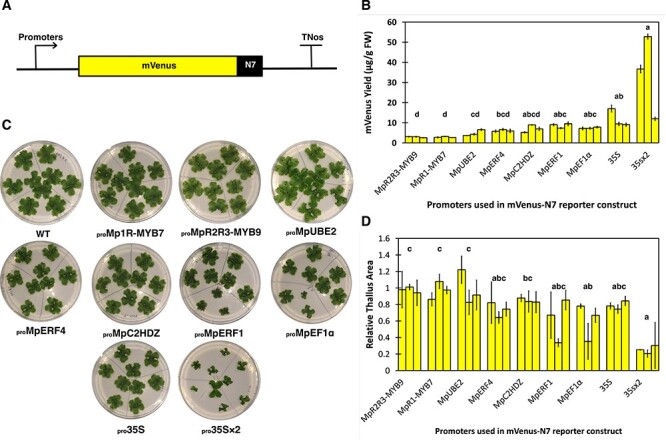
Expression level of mVenus-N7 in different promoter lines and relative size estimation of plants from different promoter:mVenus-N7 lines. (A) Schematic representation of the promoter:mVenus-N7 constructs. (B) mVenus-N7 expressed in different promoter lines in 3-week-old plants, in µg/g FW. (C) Images of 2- week-old WT and different promoter line plants grown in 9-cm Petri dishes 0.5× Gamborg B-5 basal medium supplemented with 0.5% (w/v) sucrose. The plates were divided into three portions, and on each portion grew three gemmae derived from the same independent transformant of that promoter construct. (D) Relative thallus size (the ratio of sizes of promoter line plants: average size of WT plants) of different promoter line plants (2-week-old). In B and D, each bar represents an independent transformant for that promoter construct, and error bars represent standard deviation between three biological replicates. Letters above the bars indicate statistically significant differences between the different promoter:mVenus-N7 lines (Dunn’s test; *P* < 0.05).

In order to compare expression levels for the candidate constitutive promoters, we quantified nuclear-localized mVenus-N7 reporter protein levels in 3-week-old plants. The constitutive _pro_*35S × 2* reported the highest protein yield: up to 52 µg/g FW, although the level of expression between different independent transformants varied, likely due to T-DNA insert number, positional effect or post-transcriptional silencing. Among the newly characterized ubiquitous promoters, _pro_Mp*ERF1* and _pro_Mp*C2HDZ* produced an average of 5–10 µg/g FW of mVenus-N7 in reporter lines, which is similar to _pro_*35S × 2*, _pro_*35S* and _pro_Mp*EF1*α reporter lines (**Fig. 2B**). Whereas _pro_Mp*R2R3-MYB9* and _pro_Mp*R2R3-MYB9* can provide lower levels of transgene expression, _pro_Mp*UBE2* has an intermediate performance.

To understand the relationship between protein yield and the potential growth burden on plants, we measured the size of 2-week-old plants. Variation between individual biological replicates and lines of the same construct can be seen (**Fig. 2C**). Lines with higher protein expression levels, such as _pro_*35S × 2* and _pro_Mp*EF1*α lines, had significantly smaller sizes (∼60% smaller than plants from other lines) at the same age compared to other plants (**Fig. 2D**), which suggested that high levels of recombinant protein accumulation are associated with growth defects.

### Subcellular localization and growth burden

**Fig. 3:**
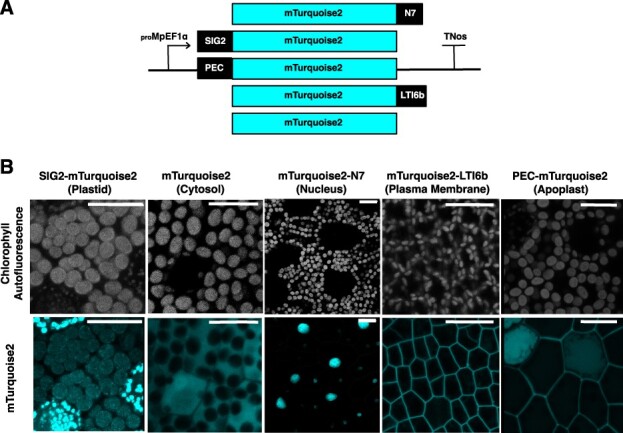
Expression pattern of mTurquoise2 reporter driven by _pro_Mp*EF1α* in day 0 Marchantia gemmae targeted to different subcellular locations. (A) Schematic representation of different subcellular targeting _pro_Mp*EF1α:mTurquoise2* constructs. N7: nuclear localization signal from the Arabidopsis ankyrin-like protein, AT4G19150. LTI6b: plasma membrane localization signal from the Arabidopsis gene AT3G05890. SIG2: chloroplast signal peptide from Marchantia SIG2 Mp4g13380.1. PEC: the N-terminal transit peptide (PEC) of an apple pectinase (GenBank accession: L27743.1). (B) Confocal microscopy images, where mTurquoise2 signal is shown light coloured and chlorophyll autofluorescence shown in gray. Scale bar = 20 µm. For labels of each construct, construct names labeled on top; intended subcellular localization is marked in bracket at the bottom.

To explore if subcellular localization of the protein product could differentially impact yield as well as growth of the transgenic plant, we tested a series of peptide fusions to drive protein accumulation to different subcellular compartments in Marchantia: apoplast, plastids, plasma membrane, nucleus and cytosol (**Fig. 3A**). The reporter mTurquoise2 was preferred over mVenus as it allowed comparison with the same reporter expressed from the chloroplast genome (Frangedakis et al. 2021). We used the plasma membrane localization signal from the Arabidopsis gene AT3G05890 (LTI6b), the nuclear localization signal from the Arabidopsis ankyrin-like protein, AT4G19150 (N7) and the chloroplast signal peptide from Marchantia SIG2 Mp4g13380 (Sauret-Güeto et al. 2020). We also adapted the N-terminal transit peptide (PEC) of an apple pectinase (GenBank accession: L27743.1) (Marillonnet et al. 2004) for apoplast targeting in Marchantia. Imaging of day 0 gemmae confirmed that the mTurquoise2 protein in all lines appeared to be located in the expected compartment (**Fig. 3B**). For _pro_Mp*EF1α:PEC-mTurquoise2* lines, we confirmed the targeting to the apoplast by plasmolysis, with the _pro_Mp*EF1α:mTurquoise2* line as a negative control (Supplementary Fig. S2). Treatment with a high level of osmoticum caused the plasma membrane and cell contents to draw away from the cell walls, seen in plasmolyzed cells containing _pro_Mp*EF1α:mTurquoise2*, whereas plasmolyzed cells from _pro_Mp*EF1α:PEC-Turquoise2* expressing gemmae showed residual fluorescence associated with the cell wall regions in the apoplast. The bulk of the fluorescent protein observed in untreated _pro_Mp*EF1α:PEC-Turquoise2* gemmae was associated with the outer part of the cell, possibly including the plasma membrane, and a portion of the PEC-mTurquoise2 protein was localized to the apoplast as intended.

**Fig. 4:**
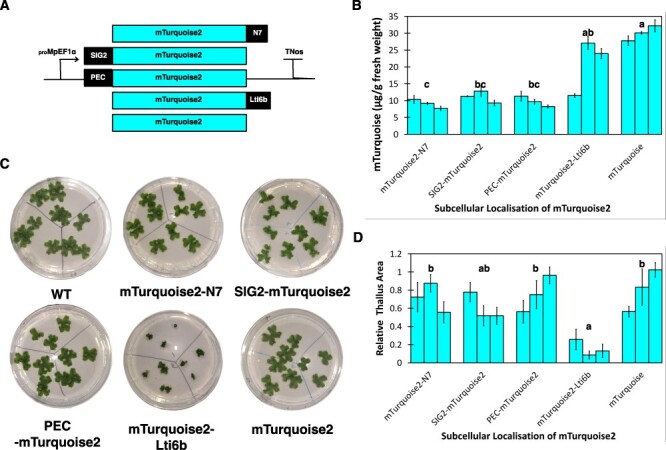
Expression level of mTurquoise2 in different subcellular-targeting lines and relative size estimation of plants from different subcellular targeting _pro_Mp*EF1α:mTurquoise2* lines. (A) Schematic representation of different subcellular targeting _pro_Mp*EF1α:mTurquoise2* constructs. (B) mTurquoise2 expressed in different subcellular-targeting lines in 3-week-old plants, in µg/g FW. (C) Images of 2-week-old WT and different subcellular-targeting line plants grown in 9-cm Petri dishes in 0.5× Gamborg B-5 basal medium. The plates were divided into three portions, and on each portion grew three gemmae derived from the same independent transformant of that promoter construct. (D) Relative thallus size (the ratio of sizes of subcellular targeting line plants: average size of WT plants) of different promoter line plants (2-week-old). In B and D, each bar represents an independent transformant for that promoter construct, and error bars represent standard deviation between three biological replicates. Letters above the bars indicate statistically significant differences between the different subcellular targeting _pro_Mp*EF1α:mTurquoise2* lines (Dunn’s test; *P* < 0.05).

We then extracted and quantified the protein yields in all lines with mTurquoise2 targeted to different cellular compartments. _pro_Mp*EF1α:mTurquoise2* lines, where mTurquoise2 is targeted to the cytosol, accumulated on average the highest amount of mTurquoise2 (∼30 µg/g FW). The average yield from _pro_Mp*EF1*α*:mTurquoise2-LTI6b* lines was the second highest at ∼25 µg/g FW, significantly higher than those for _pro_Mp*EF1*α*:mTurquoise2-N7*, _pro_Mp*EF1*α*:SIG2-mTurquoise2* and _pro_Mp*EF1*α*:PEC-mTurquoise2-LTI6b* (all ∼10 µg/g FW). Estimation of the sizes of 2-week-old plants showed that _pro_Mp*EF1α:mTurquoise2-LTI6b* plants were significantly smaller (∼70%) than all other plants apart from _pro_Mp*EF1α:SIG2-mTurquoise2* plants (**Fig. 4C, D**). This suggests that in Marchantia, the cytosol appears to be the best-performing compartment for mTurquoise2 production, while targeting recombinant protein to the plasma membrane accrued more growth penalties than retaining the protein in the cytosol.

To test the combined effects of a strong promoter and cytosolic targeting of the reporter protein on the growth of transgenic plants, we employed the promoter that allowed the highest accumulation of mVenus-N7 (_pro_*35S × 2*) with the subcellular localization that gives the highest mTurquoise2 yield (cytosol) to generate a _pro_*35S × 2:mTurquoise2* line and compare its performance with a _pro_Mp*EF1*α:mTurquoise2 line. We confirmed that the _pro_35S × 2:mTurquoise2 plants have a significantly higher (∼40%) mTurquoise2 yield, yet their sizes were similar to _pro_Mp*EF1α:mTurquoise2* plants. Cytosolic targeting allows high reporter protein yields while minimizing negative impacts on plant growth (Supplementary Fig. S3).

### Engineering betalain biosynthesis in *Marchantia*

**Fig. 5:**
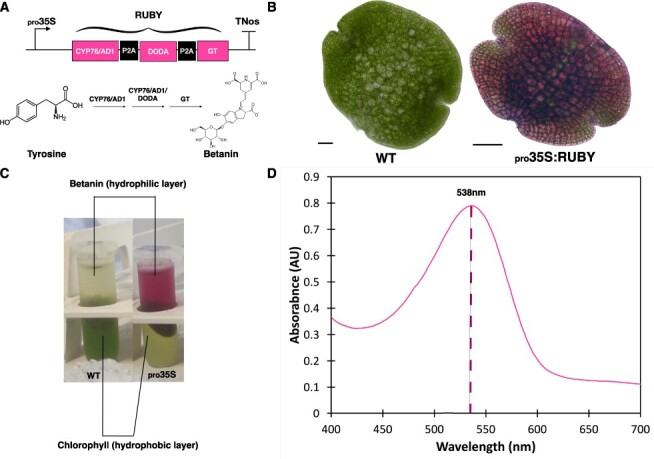
Confirmation of production of betanin by expression of the *RUBY* cassette using _pro_*35S*. (A) Schematic representation of the _pro_*35S:RUBY* construct and the simplified betanin biosynthetic pathway. (B) Bright-field microscopy images of a WT gemma and a _pro_*35S:RUBY* gemma. Scale bar = 50 µm. (C) Extraction of chlorophyll pigments (hydrophobic layer) and betanin pigments (hydrophilic layer) from 3-week-old WT and _pro_35S:RUBY plants. (D) Absorbance spectra of the extracted betalain pigments from _pro_*35S:RUBY* plants. The absorbance at 538-nm for betanin (the most prominent betalain pigment) is indicated with a dashed line.

Fluorescent proteins are very useful to visualize and measure protein accumulation in prototype systems, but are not a direct model for expression and performance of multienzyme pathways and product accumulation. As an additional test, we used six promoters: _pro_*35S*, _pro_Mp*R2R3-MYB9*, _pro_Mp*ERF1*, _pro_Mp*UBE2*, _pro_*35S × 2* and _pro_Mp*EF1*α to express the *RUBY* cassette. Betalains are a group of plant pigments synthesized from the amino acid tyrosine. They include betanin, which can be used as a bright red food colorant (Grützner et al. 2021). The *RUBY* cassette contains the coding sequences for P450 oxygenase CYP76AD1 (CYP76/AD1), L-DOPA 4,5-dioxygenase (DODA) and glucosyltransferase (GT), three enzymes required for betanin synthesis from beetroot (*Beta vulgaris*) separated by the P2A peptide (He et al. 2020) (**Fig. 5A**), a viral sequence that allows expression of multiple genes in polycistronic vectors by ribosome ‘skipping’ (de Felipe et al. 2010). For these enzymes, CYP76/AD1 is localized to the ER in yeast (DeLoache et al. 2015), and DODA and GT are localized to the cytoplasm/nucleus in plants (Chen et al. 2017). This cassette has been expressed successfully in many species (Zhao 2023).

We first demonstrated that the RUBY cassette is functional in Marchantia and produced betanin. Light microscopy of day 0 gemmae from the _pro_*35S:RUBY* line confirmed betanin accumulation, indicated by the presence of the signature bright red color (**Fig. 5B**). The absorption peak at 538-nm for extracts from the _pro_*35S:RUBY* line confirmed that the pigment produced was betanin (**Fig. 5D**).

**Fig. 6:**
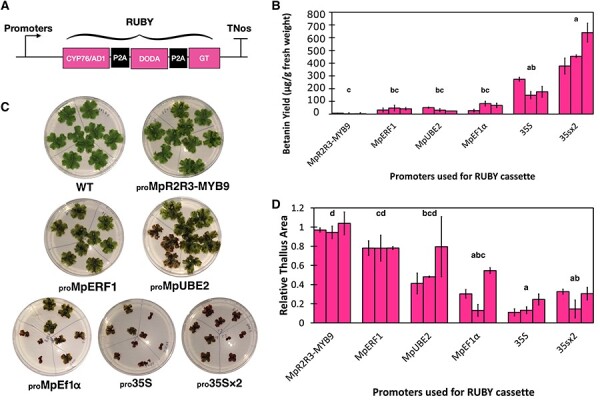
Level of betanin accumulation in different promoter:RUBY lines and relative size estimation of plants from different promoter:RUBY lines. (A) Schematic representation of different promoter:RUBY constructs. (B) Betanin extracted in different promoter:RUBY lines in 3-week-old plants, in µg/g FW. (C) Images of 2-week-old WT and different promoter:RUBY plants grown in 9-cm Petri dishes in 0.5× Gamborg B-5 basal medium supplemented with 0.5% (w/v) sucrose. The plates were divided into three portions, and on each portion grew three gemmae derived from the same independent transformant of that promoter construct. (D) Relative thallus size (the ratio of sizes of subcellular-targeting line plants: average size of WT plants) of different promoter:RUBY plants (2-week-old). In B and D, each bar represents an independent transformant for that promoter construct, and error bars represent standard deviation between three biological replicates. Letters above the bars indicate statistically significant differences between different promoter:RUBY lines (Dunn’s test; P < 0.05).

In addition to _pro_*35S*, we expressed the *RUBY* cassette in Marchantia with five other constitutive promoters: _pro_Mp*R2R3-MYB9*, _pro_Mp*ERF1*, _pro_Mp*UBE2*, _pro_*35S × 2* and _pro_Mp*EF1*α. The yields show the same pattern observed with the mVenus reporter lines. A maximum amount of ∼640 µg/g FW of betanin was obtained in 3-week-old _pro_*35S × 2:RUBY* plants, significantly higher than all other lines except _pro_*35S:RUBY* (**Fig. 6B**). The yields of betanin were negatively correlated with the size of 2-week-old plants (**Fig. 6D**), similar to the effects of overexpression seen in mVenus-N7 lines.

**Fig. 7:**
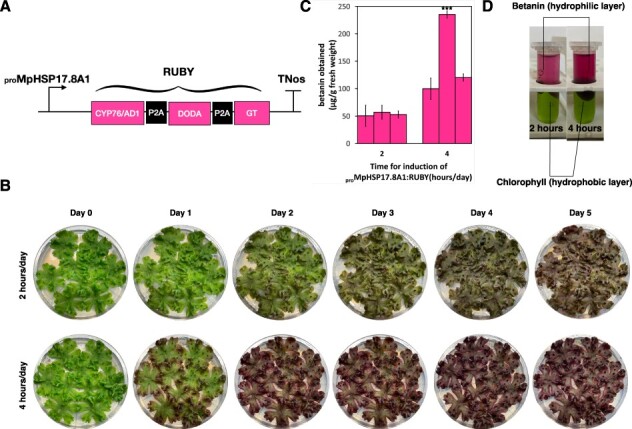
Heat-shock induction of betanin accumulation in _pro_Mp*HSP17.8A1:RUBY* lines. (A) Schematic representation of the _pro_Mp*HSP17.8A1:RUBY* construct. P2A: ribosome skipping domain. (B) The change in thallus color in 3-week-old plants from _pro_Mp*HSP17.8A1:RUBY* lines with 2 or 4 h of induction at 37°C across 5 d. The plants were grown in 9-cm Petri dishes in 0.5× Gamborg B-5 basal medium supplemented with 0.5% (w/v) sucrose. The plates were divided into three portions, and three gemmae derived from the same independent transformant of that promoter construct were grown on each segment. (C) Betanin yields from _pro_Mp*HSP17.8A1:RUBY* lines with different induction times in 3-week-old plants post-induction, in µg/g FW. Each bar represents an independent transformant for that promoter construct, and error bars represent standard deviation between three biological replicates. The triple asterisks above the bars indicate statistically significant differences between plant groups with different induction times (Wilcoxon signed-rank test, P < 0.01). (D) Extraction of chlorophyll pigments (hydrophobic layer) and betanin pigments (hydrophilic layer) from 3-week-old _pro_Mp*HSP17.8A1:RUBY* lines with 2 and 4 h of induction at 37°C.

To reduce the adverse effect of betanin production on plant growth, we drove expression of *RUBY* under the Marchantia native heat-shock promoter _pro_Mp*HSP17.8A1* (Nishihama et al. 2016). This allows the production of betanin once the plants have accumulated enough biomass (Dugdale et al. 2013). To do so, the plants were placed in a 37°C incubator for 2 and 4 h per day for 5 d and then harvested for betanin extraction and quantification. Plants with 4-h induction produced significantly higher amount of betanin (**Fig. 7**) and yielded ∼250 µg betanin/g FW, similar to _pro_*35S* lines although lower than that obtained from _pro_*35S × 2* lines.

## Discussion

The model liverwort *M. polymorpha* is a simple haploid plant with fast growth, short generation times and prolific capacity for reproduction. This combination with facile molecular genetic tools and easy access for high-resolution optical imaging make Marchantia an ideal test bed for reprogramming plant genetic systems. Promoters are essential for modulating plant gene expression, and different levels of expression are required for construction of different genetic circuits, and for reprogramming metabolism. To date, only few promoters are well characterized for their expression pattern in Marchantia, and their properties have not been well compared.

We have screened a new set of high-level, widely expressed promoters and localization tags for safe accumulation of proteins and metabolites. In this study, we expanded the promoter repertoire by characterizing five new constitutive transcription factor promoters with various levels of expression across many cell types. These provide new resources for research in Marchantia, similar to other model systems, where a spectrum of promoters with different expression levels have been made available, such as *Escherichia coli* (Anderson collection of promoters at the Registry of Standard Biological Parts), *A. thaliana* (Jores et al. 2021) and *N. benthamiana* (Tian et al. 2022).

Protein targeting to various subcellular locations was previously explored in Marchantia (Sauret-Güeto et al. 2020, Westermann et al. 2020). However, little is known of each compartment’s ability to accumulate the maximum amount of recombinant protein. We compared the yield of mTurquoise2 in various subcellular targeting lines and showed that it accumulated to the highest level when retained in the cytosol. This is at odds with some reports of recombinant protein work in flowering plants, where compartmentalization to different organelles can lead to higher recombinant protein yield (Habibi et al. 2017). This may reflect differing properties of the target protein or plant system. General principles observed in flowering plant models often do not necessarily apply to bryophytes (Niederau et al. 2024).

We used Marchantia promoters to drive expression of the recently published *RUBY* cassette as an example of producing enzymes for metabolic engineering. The beetroot betanin synthesis genes (He et al. 2020) were expressed without codon optimization, commonly required for efficient translation of heterologous proteins (Webster et al. 2017). This demonstrates the feasibility of expressing foreign metabolic pathway genes in Marchantia. 2A peptides, which allow polycistronic expression by ribosome-skipping in plants (Khosla et al. 2020) and other systems (Liu et al. 2017), have been shown to work in Marchantia for expression of two fluorescent proteins in a polycistronic vector (Waller et al. 2023). We have confirmed that the same mechanism can be applied to metabolic pathway enzymes, such as those in the betanin synthesis pathway.

High levels of transgene expression are known to have physiological consequences for hosts, and termed as ‘metabolic burden’ (Wu et al. 2016). The allocation of resources for normal host metabolism can be altered due to the expression of transgene and accumulation of recombinant proteins/metabolic products, leading to adverse effects on growth. The phenomenon was observed in tobacco chloroplast transformants that expressed high levels of recombinant proteins (Lössl et al. 2005, Hennig et al. 2007), and in Marchantia where up to 50% reduction in biomass was observed in chloroplast-engineered plants expressing mTurquoise2 up to ∼400–500 µg/g FW (Frangedakis et al. 2021). We have made similar observations for plants expressing mVenus-N7 and RUBY driven by _pro_35S × 2, where plants accumulate significantly lower biomass compared to wild type (WT) plants. However, the same is not true for the cytoplasmic _pro_*35S × 2:mTurquoise2* lines, where the transformants have similar growth rates to WT plants. This suggests that while high level of expression tends to lead to heavier metabolic burden, the exact effect is product-dependent and should be judged on a case-by-case basis. In addition, while _pro_Mp*EF1α:mTurquoise2-Lti6b* plants also accumulated high levels of mTurquoise2 similar to _pro_Mp*EF1α:mTurquoise2* plants, their growth was severely hindered, resulting in significantly smaller plants compared to other lines. This suggests that subcellular localization of recombinant proteins can affect the extent of burden seen in transgenic plants.

Despite significant suppression of growth, the maximum yield obtained in 3-week _pro_*35S × 2:RUBY* plants was 1.5 times higher than that reported in the native producer beetroot (∼300–400 µg/g FW). Slow growth due to high transgene expression was overcome by using an inducible heat-shock promoter _pro_Mp*HSP17.8A1*. The plants produced similar yields of betanin as the 3-week-old _pro_35S lines, which have one of the highest accumulations of betanin among the lines. This served as a proof-of-principle for the potential of inducible promoters to drive expression of recombinant protein for metabolic engineering in Marchantia. Furthermore, Marchantia provides a facile platform for cell and tissue selective expression and programming of synthetic source–sink relationships for safer high-level production of metabolites.

As a point of reference, the ∼60 µg/g FW of mTurquoise2 protein obtained from nuclear transformation compares with 400–500 µg/g FW of mTurquoise2 obtained from chloroplast genome transformation (Frangedakis et al. 2021). In addition, variation in yields was observed between nuclear transformed lines (**Figs. 2B**, **4B**, **6B**). This could be due to post-transcriptional RNA silencing (Matsuo and Atsumi 2019) or random insertion of the transgene cassette into the plant genome by *A. tumefaciens* (Dong and Ronald 2021). In contrast, chloroplast transformation occurs by homologous recombination into a defined chromosomal site, and gene silencing is effectively non-existent in chloroplasts. Chloroplast transformation may be the method of choice for expression of heterologous proteins, but more time consuming and less efficient than nuclear transformation (Sauret-Güeto et al. 2020), and not even feasible for many plant species. Nuclear transformation offers more potential for inducible and tissue-specific gene expression, and it may be possible to copy natural systems, and explore specialized cell types such as trichomes and oil body cells for safe storage of metabolites at very high levels.

Apart from inducible promoters, such as the heat-shock promoter _pro_Mp*HSP17.8A1* used in this study, more sophisticated genetic circuits can also be used to overcome metabolic burdens and improve heterologous expression. Genetic circuits driving gene expression under the control of multiple input signals and tissue-type-specific promoters have been implemented in plants (Brophy et al. 2022, Vazquez-Vilar et al. 2023) The combination of synthetic transcription factors, activator and repressor domains, tunable promoters (Cai et al. 2020) can achieve a better dynamic range of expression, with a completely silent OFF state and a hyperactive ON state. In Marchantia, the synthetic transcription factor GAL4-VP16 has been used (Nishihama et al. 2016), and it can potentially be used to construct orthogonal genetic circuits for enhancing recombinant protein production. Tissue-specific promoters have a great potential for agriculture and bioproduction, targeting specific tissues or cells that could be able to direct production to harvestable organs or specialized cells (Tansley et al. 2024). Similar to trichomes in vascular plants, oil body cells have potential for cell-type-specific metabolic engineering of cytotoxic terpenoids with some already well-characterized promoters (Romani et al. 2022).

Overall, we present the Marchantia research community with new promoter parts to assist gene expression research with reference data, comparing their relative strength and potential for recombinant protein production and metabolic engineering.

## Materials and Methods

### Plant material and growth conditions

*Marchantia polymorpha subs. rudelaris* accessions Cam-1 (male) and Cam-2 (female) were used (Delmans et al. 2017). Under normal conditions, plants were grown on solid 0.5× Gamborg B-5 basal medium (Phytotech #G398) at pH 5.7–5.8 with 1.2% (w/v) agar micropropagation grade (#A296 PhytoTech Labs, Lenexa, Kansas, USA) under continuous light at 21°C with a light intensity of 150 μmol/m^2^/s. The media were supplemented with 0.5% (w/v) sucrose where specified. For spore production, plants were grown in Microbox micropropagation containers (SacO_2_) in long day conditions (16 h light/8 h dark) under light supplemented with far-red light as previously described (Sauret-Güeto et al. 2020).

### Plasmid assembly

L0 DNA parts and mVenus constructs were obtained from the OpenPlant tool kit, described in (Sauret-Güeto et al. 2020) and (Romani et al. 2024), except L0-mTurquoise2 which was built by polymerase chain reaction using Phusion polymerase with the OpenPlant tool kit mTurquoise2-CDS12 part as template, with primers non-optimized mTurq2 F and non-optimized mTurq2 R. *35S:RUBY* was a gift from Yunde Zhao (Addgene plasmid #160,908; RRID:Addgene_160908; http://n2t.net/addgene:160908) (He et al. 2020), and was used as a template for creating the L0-RUBY-CDS part using a protocol described previously (Sauret-Güeto et al. 2020). The L0-RUBY-CDS part was confirmed by Sanger sequencing using primers pUAP-F, pUAP-R, as well as RUBY-1,2,3. For the assembly of L3 plasmids, a new L3 acceptor (pBy_10) was built and the plasmid map is provided in the supplementary data as a GenBank file. mTurquoise2 and *RUBY* constructs were made by one-step Type-IIS cloning of L0 parts into the L3 acceptor pBy_10 using BsaI and T4 ligase.

For the mVenus promoter constructs, the presence of the correct insert was confirmed by restriction *Xho*I digestion (#FD0694 Thermo Fisher Scientific, UK) and by Sanger sequencing using primer Rv5. For mTurquoise2 constructs, the presence of the correct insert was confirmed by *Nde*I digestion (Thermo Scientific #FD0583), and by Sanger sequencing using primers Ef1a-seq-2 and Nos-35S-seq-2. L3-RUBY constructs were confirmed by *Xho*I digestion and Sanger sequencing using the primer RUBY-seq-4.

### Agrobacterium-mediated transformation

Marchantia spores were sterilized as previously described (Sauret-Güeto et al. 2020). A modification of the published Agrobacterium-mediated protocol for transformation in six-well plates (Sauret-Güeto et al. 2020) was used, as described previously (Romani et al. 2024).

### Laser scanning confocal microscopy

For mVenus lines, images were acquired on a Leica SP5 confocal microscope upright system equipped with an Argon ion gas laser with emitted wavelengths of 458, 476, 488 and 514 nm and a

405 nm diode laser, a 594 nm HeNe laser, a 633 nm HeNe laser and a 561 DPSS laser. Imaging was conducted either using a 10× air objective (HC PL APO 675 10×/0.40 CS2) or a 2.5× air objective (HC PL APO 20×/0.75 CS2). Sequential scanning mode was selected for observing fluorescent proteins with overlapping emission spectra. Excitation laser wavelengths and emission fluorescence bandwidth windows were as follows: for mVenus (514 nm, 527– 552 nm), for mScarlet (561 nm, 595– 620 nm) and for chlorophyll autofluorescence (633 nm, 687– 739 nm).

For mTurquoise2 lines, images were acquired on an upright Leica SP8X confocal microscope equipped with a 460–670 nm supercontinuum white light laser, two continuous wave laser lines of 405 nm and 442 nm and a five-channel spectral scanhead (four hybrid detectors and one photomultiplier). Imaging was conducted using either a 20× air objective (HC PL APO 20×/0.75 CS2) or a 40× water immersion objective (HC PL APO 40×/1.10 W CORR CS2). Excitation laser wavelength and fluorescence emission bandwidth windows were as follows: 442 nm and 460–485 nm (for mTurquiose2) and 488 or 515 nm and 670–700 nm (for chlorophyll autofluorescence). Chlorophyll autofluorescence was imaged simultaneously with mTurquoise2.

### Protein extraction and yield estimation

mVenus and mTurquoise2 standard curves (random fluorescence unit against known protein concentration) were built as previously described using bacterial-expressed mVenus-7xHis-tagged and mTurquoise2-6xHis-tagged proteins (Frangedakis et al. 2021) for the estimation of the quantity of each fluorescent protein expressed in plant samples.

Marchantia thallus tissue (200 mg) from 3-week-old plants was ground on ice with a mortar and pestle and resuspended in 700 μl of protein extraction buffer [50 mM Tris-HCl, pH 7.5, 150 mM NaCl, Tween 20 0.1% (v/v), 10% (v/v) glycerol, 1 mM dithiothreitol (DTT)] plus Roche cOmplete protease inhibitor (#11836170001, Roche Diagnostics, Mannheim, Germany). Plant debris was pelleted by centrifugation (15 min, 15,000 r.p.m., 4°C), and the supernatant was treated as the total soluble protein fraction. Three replicates of 20 µl of extract for each sample were used for measuring fluorescence in a Sterilin™ Clear Microtiter™ Plate (#611V96, Thermo Fisher Scientific, UK). A BMG CLARIOstar plate reader was used with an excitation and emission wavelength appropriate for mTurquoise2 measurement (excitation: 430–20 nm, emission: 474–20 nm, gain 500 nm). Sample values were adjusted by subtracting the fluorescence values of the blank.

### Estimation of plant sizes

To estimate plant sizes and growth, the projection area was calculated using overhead pictures of Petri dishes containing 12 (three biological replicates per independent transgenic line) 2-week-old Marchantia plants. Pictures were scaled and the projected area of each individual plant was manually selected and measured using the Measure tool in ImageJ/Fiji (Schneider et al. 2012). For plots, data were normalized to the ratio of the thallus area of transgenic plants to WT plants.

### Betanin extraction and quantification

The betanin extraction method was adapted from a previously published protocol (Chang et al. 2021). Briefly, 200 mg of 3-week-old plant tissue was placed into a 2-ml Eppendorf tube with a stainless-steel bead (3–7 mm diameter), flash-frozen in liquid nitrogen, and subjected to disruption for two cycles of 1 min at 30 Hz on a TissueLyser II (Qiagen, Manchester, UK) at 4°C. Betanin pigments were then extracted with 2 ml of extraction solution [methanol:chloroform:water (1:2:1), supplemented with 1 mM of ascorbic acid], followed by 10 s of vigorous vortexing, followed by centrifugation (2 min, 4°C, 13,000 r.p.m.). After centrifugation, triplicates of 100 µl each from the upper (hydrophilic) layer were collected and dispensed into a black-walled 96-well tissue culture treated plate with a lid (#655090 Greiner Bio-One, Stonehouse, UK). A BMG CLARIOstar plate reader was used for measurement of absorbance across the full spectrum (220–975 nm) and at three particular wavelengths [538 nm for betanin absorbance, 900 nm and 975 nm for correction of path length (Lampinen et al. 2012)].

### Induction of heat-inducible promoter lines

Three gemmae from each of the three independent transformants with the heat-inducible promoter-driven RUBY cassette were grown on Gamborg B-5 plates for 3 weeks. The plants were placed in a 37°C incubator without light for 2 or 4 h each day at the same time, respectively, before being moved back to the standard growth conditions. After 5 d, plants were harvested and flash-frozen in liquid nitrogen, before being subjected to the betanin extraction protocol above.

### Data handling

Data were expressed as the means ± standard deviation. Statistical analysis was performed using R Statistical Software (R Core Team 2023). Differences between two groups were assessed using a Wilcoxon signed-rank test. For multiple comparisons, significance analysis was determined by Kruskal–Wallis one-way analysis of variance, followed by a post hoc Dunn’s test (Bonferroni correction for the final *P*-value) using the R FSA package (Ogle et al. 2023). Letter summaries of statistical similarities and differences were compiled using the R multcompView (Graves et al. 2023) and rcompanion packages (Mangiafico 2023).

## Supplementary Material

pcae063_Supp

## Data Availability

Promoter sequences are available at https://mpexpatdb.org (Romani et al. 2024). All primer sequences are provided in the supplementary data.

## References

[R1] Althoff F., Kopischke S., Zobell O., Ide K., Ishizaki K., Kohchi T., et al. (2014) Comparison of the MpEF1α and CaMV35 *promoters for application in Marchantia polymorpha* overexpression studies. *Transgenic Res*. 23: 235–244. 24036909 10.1007/s11248-013-9746-z

[R2] Amack S.C. and Antunes M.S. (2020) CaMV35S promoter – a plant biology and biotechnology workhorse in the era of synthetic biology. *Curr. Plant Biol*. 24: 100179.

[R3] Bankar S.B., Bule M.V., Singhal R.S. and Ananthanarayan L. (2009) Glucose oxidase—an overview. *Biotechnol. Adv*. 27: 489–501. 19374943 10.1016/j.biotechadv.2009.04.003

[R4] Bowman J.L., Arteaga-Vazquez M., Berger F., Briginshaw L.N., Carella P., Aguilar-Cruz A., et al. (2022) The renaissance and enlightenment of *Marchantia* as a model system. *Plant Cell* 34: 3512–3542. 35976122 10.1093/plcell/koac219 PMC9516144

[R5] Brophy J.A.N., Magallon K.J., Duan L., Zhong V., Ramachandran P., Kniazev K., et al. (2022) Synthetic genetic circuits as a means of reprogramming plant roots. *Science* 377: 747–751. 35951698 10.1126/science.abo4326

[R6] Brückner K., Schäfer P., Weber E., Grützner R., Marillonnet S., and and Tissier A. (2015) A library of synthetic transcription activator-like effector-activated promoters for coordinated orthogonal gene expression in plants. *Plant J*. 82: 707–716. 25846505 10.1111/tpj.12843 PMC4691316

[R7] Cai Y.-M., Kallam K., Tidd H., Gendarini G., Salzman A. and Patron N. (2020) Rational design of minimal synthetic promoters for plants. *Nucleic Acids Res*. 48: 11845–11856. 32856047 10.1093/nar/gkaa682 PMC7708054

[R8] Chang Y.-C., Chiu Y.-C., Tsao N.-W., Chou Y.-L., Tan C.-M., Chiang Y.-H., et al. (2021) Elucidation of the core betalain biosynthesis pathway in Amaranthus tricolor. *Sci. Rep*. 11: 6086. 10.1038/s41598-021-85486-x PMC7969944 33731735

[R9] Chen N., Yu Z.-H. and Xiao X.-G. (2017) Cytosolic and nuclear co-localization of betalain biosynthetic enzymes in tobacco suggests that betalains are synthesized in the cytoplasm and/or nucleus of betalainic plant cells. *Front. Plant Sci*. 8: 242166. 10.3389/fpls.2017.00831 PMC5435750 28572813

[R10] de Felipe P., Luke G.A., Brown J.D. and Ryan M.D. (2010) Inhibition of 2A-mediated ‘cleavage’ of certain artificial polyproteins bearing N-terminal signal sequences. *Biotechnol. J*. 5: 213–223. 19946875 10.1002/biot.200900134 PMC2978324

[R11] Delmans M., Pollak B. and Haseloff J. (2017) MarpoDB: an open registry for *Marchantia polymorpha* genetic parts. *Plant Cell Physiol*. 58: e5. 10.1093/pcp/pcw201 PMC5444569 28100647

[R12] DeLoache W.C., Russ Z.N., Narcross L., Gonzales A.M., Martin V.J.J. and Dueber J.E. (2015) An enzyme-coupled biosensor enables (S)-reticuline production in yeast from glucose. *Nat. Chem. Biol*. 11: 465–471. 25984720 10.1038/nchembio.1816

[R13] Dong O.X. and Ronald P.C. (2021) Targeted DNA insertion in plants. *Proc. Natl. Acad. Sci. USA* 118: e2004834117. 10.1073/pnas.2004834117 PMC8179203 34050013

[R14] Dugdale B., Mortimer C.L., Kato M., James T.A., Harding R.M. and Dale J.L. (2013) In plant activation: an inducible, hyperexpression platform for recombinant protein production in plants. *Plant Cell*. 25: 2429–2443. 23839786 10.1105/tpc.113.113944 PMC3753375

[R15] Engler C., Youles M., Gruetzner R., Ehnert T.-M., Werner S., Jones J.D.G., et al. (2014) A golden gate modular cloning toolbox for plants. *ACS Synth. Biol*. 3: 839–843. 24933124 10.1021/sb4001504

[R16] Feng Z., Li X., Fan B., Zhu C., and Chen Z. (2022) Maximizing the production of recombinant proteins in plants: from transcription to protein stability. *Int. J. Mol. Sci*. 23: 13516. 10.3390/ijms232113516 PMC9659199 36362299

[R17] Finka A., Saidi Y., Goloubinoff P., Neuhaus J.-M., Zrÿd J.-P. and Schaefer D.G. (2008) The knock-out of ARP3a gene affects F-actin cytoskeleton organization altering cellular tip growth, morphology and development in moss Physcomitrella patens. *Cell Motil. Cytoskeleton* 65: 769–784. 18613119 10.1002/cm.20298

[R18] Frangedakis E., Guzman-Chavez F., Rebmann M., Markel K., Yu Y., Perraki A., et al. (2021) Construction of DNA tools for hyperexpression in *Marchantia* chloroplasts. *ACS Synth. Biol*. 10: 1651–1666. 34097383 10.1021/acssynbio.0c00637 PMC8296666

[R19] Gils M., Kandzia R., Marillonnet S., Klimyuk V. and Gleba Y. (2005) High‐yield production of authentic human growth hormone using a plant virus‐based expression system. *Plant Biotechnol. J*. 3: 613–620. 17147632 10.1111/j.1467-7652.2005.00154.x

[R20] Graves S., Piepho H.-P. and Selzer L. (2023) Visualizations of paired comparisons. https://cran.r-project.org/web/packages/multcompView/multcompView.pdf (December 10, 2023, date last accessed).

[R21] Grützner R., Schubert R., Horn C., Yang C., Vogt T. and Marillonnet S. (2021) Engineering betalain biosynthesis in tomato for high level betanin production in fruits. *Front. Plant Sci*. 12: 682443. 10.3389/fpls.2021.682443 PMC8220147 34177999

[R22] Habibi P., Prado G.S., Pelegrini P.B., Hefferon K.L., Soccol C.R. and Grossi-de-sa M.F. (2017) Optimization of inside and outside factors to improve recombinant protein yield in plant. *Plant Cell Tissue Organ Cult*. 130: 449–467.

[R23] Han Y.-J., Kim Y.-M., Hwang O.-J. and Kim J.-I. (2015) Characterization of a small constitutive promoter from *Arabidopsis* translationally controlled tumor protein (AtTCTP) gene for plant transformation. *Plant Cell Rep*. 34: 265–275. 25410250 10.1007/s00299-014-1705-5

[R24] He Y., Zhang T., Sun H., Zhan H. and Zhao Y. (2020) A reporter for noninvasively monitoring gene expression and plant transformation. *Hortic. Res*. 7: 152. 10.1038/s41438-020-00390-1 PMC7502077 33024566

[R25] Hennig A., Bonfig K., Roitsch T. and Warzecha H. (2007) Expression of the recombinant bacterial outer surface protein A in tobacco chloroplasts leads to thylakoid localization and loss of photosynthesis. *FEBS J*. 274: 5749–5758. 17922845 10.1111/j.1742-4658.2007.06095.x

[R26] Holtorf S., Apel K. and Bohlmann H. (1995) Comparison of different constitutive and inducible promoters for the overexpression of transgenes in *Arabidopsis thaliana*. *Plant Mol. Biol*. 29: 637–646. 8541491 10.1007/BF00041155

[R27] Ishizaki K., Chiyoda S., Yamato K.T. and Kohchi T. (2008) *Agrobacterium*-mediated transformation of the haploid liverwort *Marchantia polymorpha* L., an emerging model for plant biology. *Plant Cell Physiol*. 49: 1084–1091. 18535011 10.1093/pcp/pcn085

[R28] Ishizaki K., Nishihama R., Ueda M., Inoue K., Ishida S., Nishimura Y., et al. (2015) Development of gateway binary vector series with four different selection markers for the liverwort *Marchantia polymorpha*. *PLoS One* 10: e0138876. 10.1371/journal.pone.0138876 PMC4583185 26406247

[R29] Jiang P., Zhang K., Ding Z., He Q., Li W., Zhu S., et al. (2018) Characterization of a strong and constitutive promoter from the *Arabidopsis* serine carboxypeptidase-like gene AtSCPL30 as a potential tool for crop transgenic breeding. *BMC Biotech*. 18: 59. 10.1186/s12896-018-0470-x PMC6151023 30241468

[R30] Jores T., Tonnies J., Wrightsman T., Buckler E.S., Cuperus J.T., Fields S., et al. (2021) Synthetic promoter designs enabled by a comprehensive analysis of plant core promoters. *Nat. Plants* 7: 842–855. 34083762 10.1038/s41477-021-00932-y PMC10246763

[R31] Khosla A., Rodriguez‐Furlan C., Kapoor S., Van Norman J.M. and Nelson D.C. (2020) A series of dual-reporter vectors for ratiometric analysis of protein abundance in plants. *Plant Direct*. 4: e00231. 10.1002/pld3.231 PMC7306620 32582876

[R32] Lampinen J., Raitio M., Perälä A. and Oranen H. (2012) Microplate based pathlength correction method for photometric DNA quantification assay. *Vantaa: Thermo Fisher Application Note*.

[R33] Liu H. and Timko M.P. (2022) Improving protein quantity and quality—the next level of plant molecular farming. *Int. J. Mol. Sci*. 23: 1326. 10.3390/ijms23031326 PMC8836236 35163249

[R34] Liu Z., Chen O., Wall J.B.J., Zheng M., Zhou Y., Wang L., et al. (2017) Systematic comparison of 2A peptides for cloning multi-genes in a polycistronic vector. *Sci. Rep*. 7: 2193. 10.1038/s41598-017-02460-2 PMC5438344 28526819

[R35] Lössl A., Bohmert K., Harloff H., Eibl C., Mühlbauer S. and Koop H.-U. (2005) Inducible trans-activation of plastid transgenes: expression of the R. eutrophaphb operon in transplastomic tobacco. *Plant Cell Physiol*. 46: 1462–1471. 15964903 10.1093/pcp/pci157

[R36] Mangiafico S.S. (2023) Rcompanion: functions to support extension education program evaluation. Rutgers Cooperative Extension, New Brunswick, New Jersey. https://CRAN.R-project.org/package=rcompanion/ (December 12, 2023, date last accessed).

[R37] Marillonnet S., Giritch A., Gils M., Kandzia R., Klimyuk V. and Gleba Y. (2004) In planta engineering of viral RNA replicons: efficient assembly by recombination of DNA modules delivered by *Agrobacterium*. *Proc. Natl. Acad. Sci. USA* 101: 6852–6857. 15103020 10.1073/pnas.0400149101 PMC406431

[R38] Matsuo K. and Atsumi G. (2019) CRISPR/Cas9-mediated knockout of the RDR6 gene in *Nicotiana benthamiana* for efficient transient expression of recombinant proteins. *Planta* 250: 463–473. 31065786 10.1007/s00425-019-03180-9

[R39] Muthamilselvan T., Kim J.S., Cheong G. and Hwang I. (2019) Production of recombinant proteins through sequestration in chloroplasts: a strategy based on nuclear transformation and post-translational protein import. *Plant Cell Rep*. 38: 825–833. 31139894 10.1007/s00299-019-02431-z

[R40] Niederau P.A., Eglé P., Willig S., Parsons J., Hoernstein S.N.W., Decker E.L., et al. (2024) Multifactorial analysis of terminator performance on heterologous gene expression in Physcomitrella. *Plant Cell Rep*. 43: 43. 10.1007/s00299-023-03088-5 PMC10800305 38246952

[R41] Nishihama R., Ishida S., Urawa H., Kamei Y., Kohchi T., et al. (2016) Conditional gene expression/deletion systems for *Marchantia polymorpha* using its own heat-shock promoter and Cre/loxP-mediated site-specific recombination. *Plant Cell Physiol*. 57: 271–280. 26148498 10.1093/pcp/pcv102

[R42] Ogle D.H., Doll J.C., Powell Wheeler A. and Dinno A. (2023) FSA: simple fisheries stock assessment methods. https://CRAN.R-project.org/package=FSA (December 10, 2023, date last accessed).

[R43] Patron N.J., Orzaez D., Marillonnet S., Warzecha H., Matthewman C., Youles M., et al. (2015) Standards for plant synthetic biology: a common syntax for exchange of DNA parts. *New Phytol*. 208: 13–19. 26171760 10.1111/nph.13532

[R44] R Core Team (2023) R: A language and environment for statistical computing. R foundation for statistical computing, Vienna, Austria. https://www.R-project.org (December 10, 2023, date last accessed).

[R45] Romani F., Flores J.R., Tolopka J.I., Suárez G., He X. and Moreno J.E. (2022) Liverwort oil bodies: diversity, biochemistry, and molecular cell biology of the earliest secretory structure of land plants. *J. Exp. Bot*. 73: 4427–4439. 35394035 10.1093/jxb/erac134

[R46] Romani F., Sauret-Güeto S., Rebmann M., Annese D., Bonter I., Tomaselli M. et al. (2024) The landscape of transcription factor promoter activity during vegetative development in *Marchantia*. *Plant Cell*. 36: 2140–2159. 38391349 10.1093/plcell/koae053 PMC11132968

[R47] Saberianfar R., Joensuu J.J., Conley A.J. and Menassa R. (2015) Protein body formation in leaves of *Nicotiana benthamiana*: a concentration-dependent mechanism influenced by the presence of fusion tags. *Plant Biotechnol. J*. 13: 927–937. 25640969 10.1111/pbi.12329

[R48] Saidi Y., Schaefer D.G., Goloubinoff P., Zrÿd J.-P. and Finka A. (2009) The CaMV 35S promoter has a weak expression activity in dark grown tissues of moss Physcomitrella patens. *Plant Signal. Behav*. 4: 457–459. 19816109 10.4161/psb.4.5.8541 PMC2676766

[R49] Sauret-Güeto S., Frangedakis E., Silvestri L., Rebmann M., Tomaselli M., Markel K., et al. (2020) Systematic tools for reprogramming plant gene expression in a simple model, *Marchantia polymorpha*. *ACS Synth. Biol*. 9: 864–882. 32163700 10.1021/acssynbio.9b00511

[R50] Schneider C.A., Rasband W.S. and Eliceiri K.W. (2012) NIH image to imageJ: 25 years of image analysis. *Nat. Methods* 9: 671–675. 22930834 10.1038/nmeth.2089 PMC5554542

[R51] Takemura M., Kanamoto H., Nagaya S. and Ohyama K. (2013) Bioproduction of prostaglandins in a transgenic liverwort, *Marchantia polymorpha*. *Transgenic Res*. 22: 905–911. 23463075 10.1007/s11248-013-9699-2

[R52] Tansley C., Patron N.J. and Guiziou S. (2024) Engineering plant cell fates and functions for agriculture and industry. *ACS Synth. Biol*. 13: 998–1005. 38573786 10.1021/acssynbio.4c00047 PMC11036505

[R53] Teh Y.-H.A. and Kavanagh T.A. (2010) High-level expression of Camelid nanobodies in *Nicotiana benthamiana*. *Transgenic Res*. 19: 575–586. 19862637 10.1007/s11248-009-9338-0

[R54] Tian C., Zhang Y., Li J. and Wang Y. (2022) Benchmarking intrinsic promoters and terminators for plant synthetic biology research. *Biodes. Res*. 2022: 9834989. 10.34133/2022/9834989 PMC10521690 37850139

[R55] Vazquez-Vilar M., Selma S., Orzaez D. and Yang J.-S. (2023) The design of synthetic gene circuits in plants: new components, old challenges. *J. Exp. Bot*. 74: 3791–3805. 37204924 10.1093/jxb/erad167 PMC10353530

[R56] Villao-Uzho L., Chávez-Navarrete T., Pacheco-Coello R., Sánchez-Timm E. and Santos-Ordóñez E. (2023) Plant promoters: their identification, characterization, and role in gene regulation. *Genes* 14: 1226. 10.3390/genes14061226 PMC10298551 37372407

[R57] Waller M., Frangedakis E., Marron A.O., Sauret‐Güeto S., Rever J., Sabbagh C.R.R., et al. (2023) An optimized transformation protocol for Anthoceros agrestis and three more hornwort species. *Plant J*. 114: 699–718. 36811359 10.1111/tpj.16161 PMC10952725

[R58] Webster G.R., Teh A.Y.-H. and Ma J.K.-C. (2017) Synthetic gene design—the rationale for codon optimization and implications for molecular pharming in plants. *Biotechnol. Bioeng*. 114: 492–502. 27618314 10.1002/bit.26183

[R59] Westermann J., Koebke E., Lentz R., Hülskamp M. and Boisson-Dernier A. (2020) A comprehensive toolkit for quick and easy visualization of marker proteins, protein–protein interactions and cell morphology in *Marchantia polymorpha*. *Front. Plant Sci*. 11: 569194. 10.3389/fpls.2020.569194 PMC7593560 33178238

[R60] Wu G., Yan Q., Jones J.A., Tang Y.J., Fong S.S. and Koffas M.A.G. (2016) Metabolic burden: cornerstones in synthetic biology and metabolic engineering applications. *Trends Biotechnol*. 34: 652–664. 26996613 10.1016/j.tibtech.2016.02.010

[R61] Yang E.J.Y. and Nemhauser J.L. (2023) Building a pipeline to identify and engineer constitutive and repressible promoters. *Quant. Plant. Biol*. 4: e12. 10.1017/qpb.2023.10 PMC10600573 37901686

[R62] Zhao Y. (2023) The RUBY reporter – Zhao lab. https://zhaolab.biosci.ucsd.edu/ruby/ (December 10, 2023, date last accessed).

